# ReactorAFM/STM – dynamic reactions on surfaces at elevated temperature and atmospheric pressure

**DOI:** 10.3762/bjnano.16.30

**Published:** 2025-03-21

**Authors:** Tycho Roorda, Hamed Achour, Matthijs A van Spronsen, Marta E Cañas-Ventura, Sander B Roobol, Willem Onderwaater, Mirthe Bergman, Peter van der Tuijn, Gertjan van Baarle, Johan W Bakker, Joost W M Frenken, Irene M N Groot

**Affiliations:** 1 Leiden Institute of Chemistry, Leiden University, Rapenburg 70, Leiden, 2311 EZ, Netherlands; 2 Leiden Institute of Physics, Leiden University, Rapenburg 70, Leiden, 2311 EZ, Netherlands; 3 ASML, Veldhoven, Netherlands; 4 Leiden Probe Microscopy, Leiden University, Rapenburg 70, Leiden, 2311 EZ, Netherlands

**Keywords:** combined AFM/STM, conductive AFM, model catalysts, nc-AFM, operando catalysis, qPlus tuning fork sensor

## Abstract

Previous work has shown the ReactorSTM and ReactorAFM, capable of studying materials under industrially relevant conditions. Here we show current developments of the ReactorAFM/STM, implementing a qPlus sensor to add the ability of combining atomic force microscopy (AFM) and scanning tunneling microscopy (STM) techniques to study the geometric and electronic structure of materials under reaction conditions. We demonstrate this by imaging a Pd(100) single crystal at 450 K with combined AFM/STM. The surface is compared under ultrahigh vacuum and under 0.5 bar O_2_ pressure showing a notable increase in RMS current, which we attribute to oxidation. Also, we study cobalt nanoparticle catalysts on an aluminum oxide support, industrially relevant in the Fischer–Tropsch synthesis. The catalysts are imaged before and after reaction at 430 K as the current maximum temperature of the qPlus sensor used falls just below the reaction temperature. Quadrupole mass spectrometry data show the reaction taking place by monitoring product gases during heating and cooling of the sample under CO and H_2_ gas pressures of 2 bar. The monitored gases include H_2_O as byproduct and the hydrocarbons ethane (*m*/*z* = 30), propane (*m*/*z* = 44), and hexane (*m*/*z* = 86), which all show increases in counts while between 490 and 550 K. The added ability to scan various surfaces with combined AFM/STM while monitoring the reaction products demonstrates the versatility offered by the ReactorAFM/STM to study catalysts under realistic industrial conditions.

## Introduction

Operando catalysis is the field of research that monitors the structure, composition, and morphology of a catalyst while simultaneously investigating its activity, reactivity, and selectivity under industrially relevant conditions. While much research has been conducted at room temperatures (or below) and under ambient to ultrahigh vacuum (UHV) conditions, industrial conditions expose catalysts to 1000 K and beyond in pressures ranging from ambient to 100 bar [1–2]. This difference in pressure, which influences whether a given catalyst becomes reactive, is referred to as the pressure gap. To provide an interpretive framework for catalysts under industrial conditions, new experimental and theoretical analysis tools are required. While recent years have witnessed a tremendous effort in this direction [3], many of these techniques are photon-based [4–9]. Even though they provide valuable insights, the development of surface-sensitive techniques that can image the catalyst at the atomic scale under high-pressure and high-temperature conditions remains crucial.

In attempting to close the pressure gap, a high-pressure ReactorSTM has been developed [10–11]. The pressures in the scanning tunneling microscopy (STM) reactor are orders of magnitude above UHV (up to several bar), rendering gas–catalyst interactions very significant and leading to differences in reaction mechanisms [12–15]. Alongside the pressure gap, the existence of the materials gap refers to the complexity and heterogeneity of real catalysts. Such catalysts are compounds that possess a special complex mixture consisting essentially of metals, oxides, and promoters, supported on refractory oxides. The requirement of a conductive substrate limits STM techniques in relevant industrial applications involving such more complex catalysts. For this reason, an atomic force microscopy (AFM) version of the high-pressure STM employing a quartz tuning fork (QTF) was introduced to overcome this limitation [16]. Unlike STM, which uses the electric tunneling effect, AFM probes the forces of the tip–sample interaction. This makes AFM independent of surface conductivity and therefore a powerful tool to bridge the materials gap. The drawback of this high-pressure AFM setup is that it could not be combined with STM. While STM provides insights of the electronic state and structure of the surface, AFM offers structural and electrostatic information. Therefore, combining AFM with STM brings the best of both techniques together and offers a more precise understanding of catalytic systems.

In this paper, we present the design of a combined AFM/STM integrated in a high-pressure gas flow reactor. The combined technique is based on the state-of-the-art tuning fork sensor in a qPlus configuration with three contacts, two for AFM and one for STM [17]. In order to illustrate the applicability of the technique, operando oxidation of Pd(100) under 0.5 bar of oxygen at 450 K was carried out using nc-AFM while simultaneously recording the electrical current signal of the formed oxide. Furthermore, we show high-temperature and high-pressure images just below our current temperature limit for an industrially relevant catalyst undergoing Fischer–Tropsch synthesis (FTS).

### Challenges while applying a tuning fork as force sensor

A drawback of the QTF as a force sensor is that its temperature sensitivity increases with increasing temperatures; in fact, it can even serve as a micro temperature sensor [18]. As a result, temperature fluctuations while scanning cause shifts in resonance frequency, which could be misinterpreted as a force signal. A QTF’s resonance frequency changes with temperature according to the following equation:


[1] $$\frac{\Delta f}{f_{0}}=1-c{\left(T-T_{0}\right)}^{2},$$

where Δ*f* is the shift in resonance frequency, *f*_0_ is the natural resonance frequency of the tuning fork (with tip glued on, at *T*_0_), *c* refers to the parabolic temperature coefficient, *T* is the temperature, and *T*_0_ refers to the maximum where the tuning fork is designed to be the least temperature-dependent. Since tuning forks have originally been mass-produced for time keeping in watches, *T*_0_ is designed to be at room temperature (RT). Using a QTF as a force sensor implies that temperature fluctuations can be interpreted as height features and ultimately results in losing contact with the surface when these fluctuations exceed the setpoint of the scanner. When scanning at temperatures farther from *T*_0_, this effect becomes larger.

The QTF’s resonance frequency depends on pressure according to the following equation:


[2] $$\frac{\Delta f}{f_{0}}=-\frac{1}{2}\frac{\mu }{\rho A},$$

where μ is the added mass due to the interaction with surrounding gas molecules, ρ is the density of the quartz tuning fork, and *A* is the area of the cross section [19]. Basically, the pressure dependence is due to dampening of the prong with respect to the gas molecules around it. While we do encounter reductions in scanning quality with increasing pressure, this effect is less prominent than the limitations caused by increased temperatures.

## Experimental setup

### Overview

**Figure 1:**
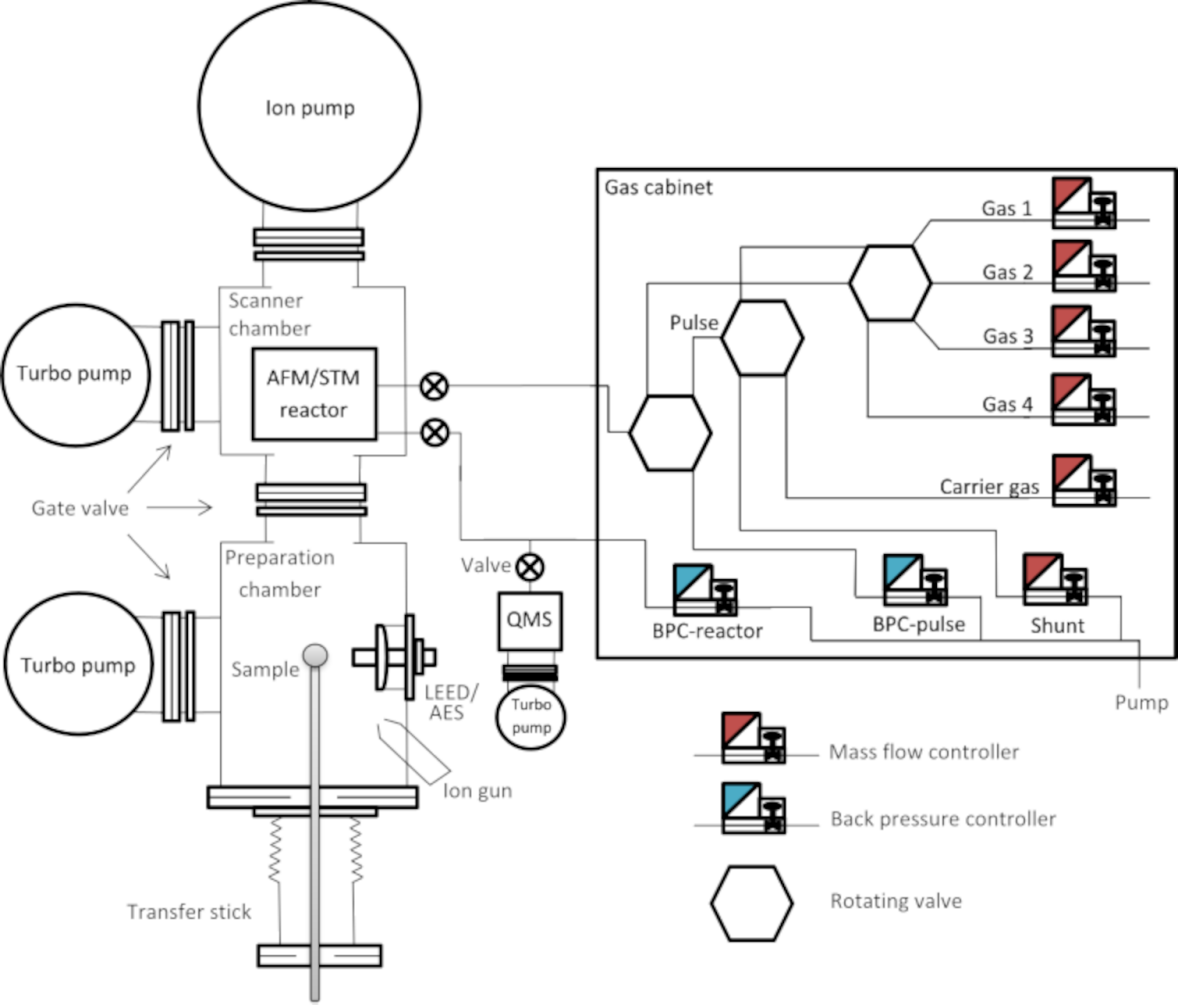
Schematic overview of the experimental setup (left side) and the gas cabinet (boxed in on the right) for gas mixing and analysis. Up to four reactive gases and a carrier gas can be mixed via a computer-controlled manifold, consisting of rotating valves, several mass flow controllers, and two back pressure controllers (BPC).

A simplified diagram of the experimental setup is shown in Figure 1. The apparatus is composed of two chambers maintained at UHV conditions and separated by a gate valve. The preparation chamber is devoted to sample preparation and characterization, while the other accommodates the AFM/STM reactor. The sample can be introduced into the setup by means of a load lock and transferred throughout the chambers with a transfer stick. The preparation chamber accommodates standard surface preparation techniques including an ion sputter gun, an e-beam evaporator, a quadrupole mass spectrometer, as well as a combined low-energy electron diffraction/Auger electron spectroscopy system to verify the cleanliness, structure, and composition of the surface. The UHV system is supported by four pneumatic air legs resting on a concrete slab, which is separated from the foundation of the building, and isolated from the main floor in an ultramicroscopy hall.

The AFM/STM is contained in a high-pressure cell and sealed off from the UHV chamber by a Kalrez elastomer seal, which exhibits outstanding thermal stability and chemical resistance together with improved sealing performance in both high-pressure and high-temperature environments. Being in direct contact with the sample surface, the seal’s maximum temperature poses a reaction temperature limitation of 600 K [11]. The vacuum is maintained by a corrosion-resistant turbo pump as well as an ion pump. A gas cabinet for gas mixing, consisting of several rotating valves, mass flow controllers, and back pressure controllers, is connected to the AFM/STM reactor, permitting pressures of up to 20 bar. Four gases plus a carrier gas can be mixed and transported to and from the reactor by capillaries at gas mixing ratios ranging from 1:1 up to 1:100 with a flow up to 40 mL/min controlled via a Python script. A separate quadrupole mass spectrometer (QMS) at the exhaust of the reactor chamber is equipped with its own turbo pump allowing for a direct correlation between surface morphology and catalytic properties by measuring the gases that leave the reactor.

### AFM/STM reactor

**Figure 2:**
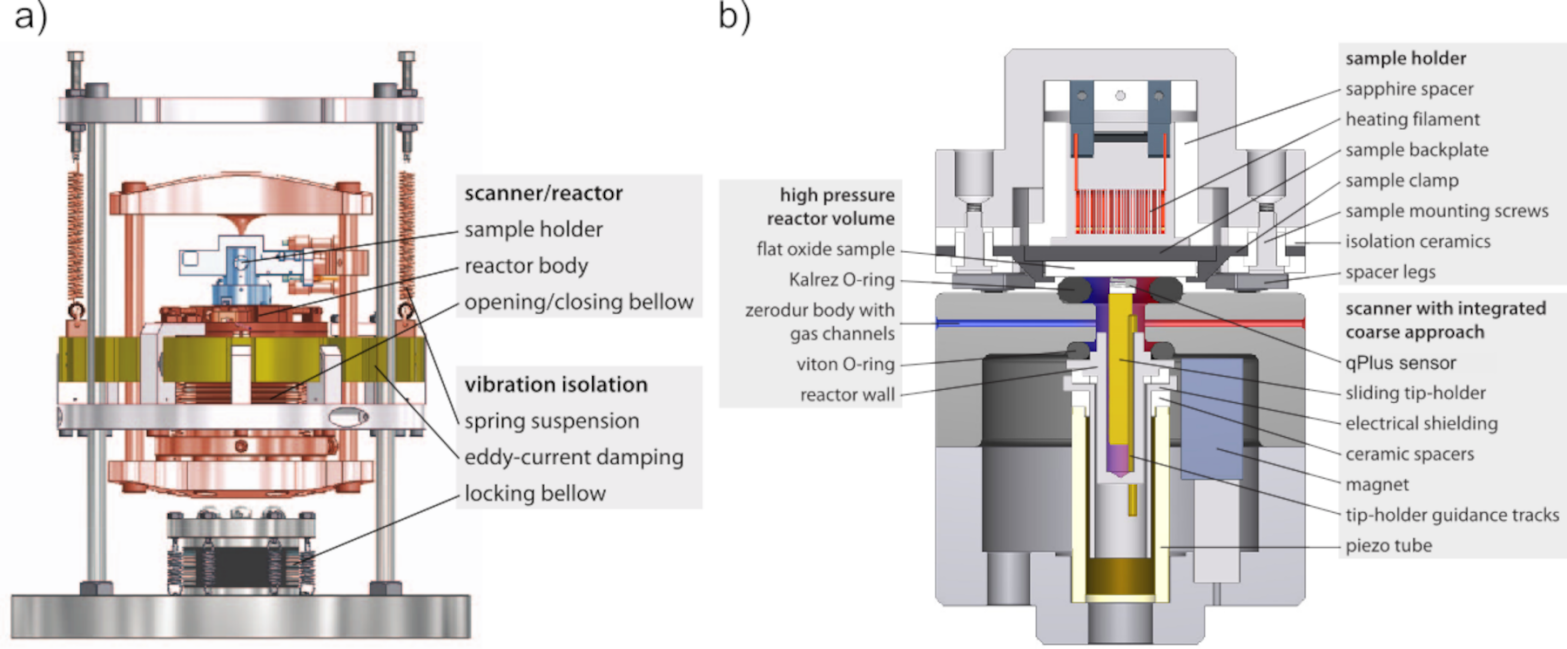
(a) Detailed schematic of the scanner assembly with vibration isolation, mounted on a CF-200 flange. Figure 2a was reproduced from [11], © 2014 C. T. Herbschieb et al., published by AIP Publishing, distributed under the terms of the Creative Commons Attribution 3.0 Generic License, https://creativecommons.org/licenses/by/3.0). (b) Detailed schematic of the ReactorAFM/STM cross section. The qPlus sensor is contained within a small high-pressure volume in the reactor body. The sample forms one side of the reactor while the remaining reactor walls are chemically inert (Zerodur). High-temperature-resistant and inert Kalrez O-rings seal off the high-pressure volume from the UHV system. Figure 2b was reproduced from [16], © 2015 S. B: Roobol et al., published by AIP Publishing, distributed under the terms of the Creative Commons Attribution 3.0 Generic License, https://creativecommons.org/licenses/by/3.0).

The main objective in the design of the AFM/STM reactor is the possibility to observe the structural and electronic properties of the surface at high gas pressures and temperatures, independent of its conductivity. The extension from STM-only and AFM-only to the combined AFM/STM reactor is an ongoing development of the existing ReactorSTM and ReactorAFM [11,16]. Therefore, in this section we will only go briefly through the main aspects of the reactor setup. The AFM/STM insert, as shown in Figure 2a,b, demonstrates the following configuration:

- The microscope (highlighted in red in Figure 2a is suspended by springs and eddy current damping for vibration isolation.
- The sample holder (highlighted in blue in Figure 2a is inserted by locking the spring mechanism with the locking bellow and then fixed to the microscope by inflating the “reactor” bellow.
- The substrate can be heated from behind by electron bombardment using a tungsten heating filament.
- The qPlus sensor is mounted to a three-contact slider and controlled by a piezotube. The piezotube is outside of the reactor volume.

Figure 2b shows a schematic cross section of the AFM/STM reactor together with the sample holder. For high-pressure experiments, the reactor volume needs to be sealed off from the UHV surrounding. This is achieved by inflating the upper bellow with pressurized air against the Kalrez O-ring, which is located on top of the reactor body sealing off the sample. This ensures that only the AFM/STM sensor and its holder are inside the reactor, while the other microscope components, such as the piezoelement, are under UHV conditions. The reactor volume is connected to two gas capillaries that transport gas in and out of the sealed reactor. The gas is then analyzed during the reaction by means of a QMS, which is directly connected to the exit channel of the reactor. In order to make the setup catalytically inert, the materials that make up both the reactor and the sample holder have been chosen to be chemically inert. For further details of the STM and AFM reactor design and performance, we refer to [16,20].

### qPlus-sensor-based AFM/STM

The core of our AFM/STM scanner incorporates a third-generation QTF in a qPlus configuration with one prong fixed and the other one carrying a metallic tip positioned at the very end [17]. The main reasons for employing this kind of sensor are its very high sensitivity to short-range forces, simultaneous acquisition of the tunneling current, and its small oscillation amplitudes (10 pm to 100 nm) [16]. The fundamental limits of the quartz tuning fork as a force sensor in scanning probe microscopy have been discussed in detail by Grober and colleagues [21]. Moreover, conventional AFM requires an optical detection method with a laser diode, which is not compatible with the design limitations of a reactor volume of 95 μL.

**Figure 3:**
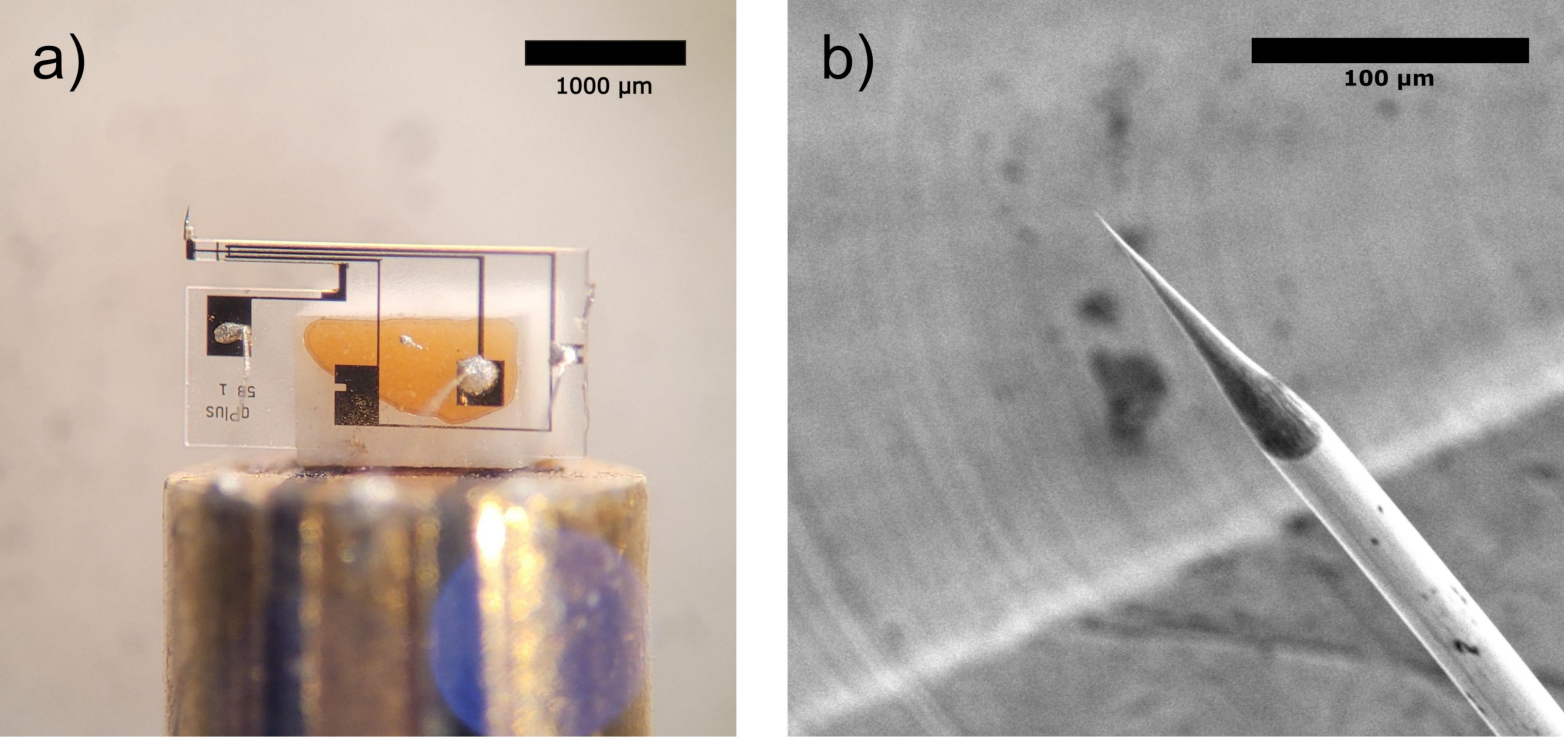
(a) M5B qPlus sensor mounted to and connected through a 3-contact slider for AFM drive, AFM readout, and STM readout. (b) Scanning electron microscopy image of a 25 μm wide chemically etched Pt/Ir wire tip.

Figure 3a shows a zoomed-in image of a third-generation M5B qPlus sensor (purchased from Nanosurf). The sensor has four gold electrodes of which three are used for AFM drive and readout, and current signal. The fourth electrode (on the back) is connected to one of the AFM contacts. The sensor is glued to a ceramic block with a non-conductive glue (EpoTek H770E), which is mounted on the slider. The slider is made of a high-speed steel rod, which is partitioned in three and isolated by non-conductive epoxy. The metallic sections are then coated with gold to ensure catalytic inertness and slide over tracks that serve as electrical feedthrough. The wiring from the QTF electrodes to the holder is made with 25 μm-diameter gold wire glued with conductive epoxy (EpoTek H20E). The holder with the QTF is magnetically held inside the piezotube that consists of a single tube and performs both the coarse approach and the fine scanning motion. The piezoelement, made of lead zirconate titanate, is placed outside the reactor and rests on an aluminum tube, which is part of the reactor wall and serves as a protection against the high-pressure gases, as well as a shield from high piezovoltages. The scanner range can go up to 3.6 μm × 3.6 μm and does not have a coarse range in the *x* and *y* directions.

### Tip preparation

Tips are fabricated by electrochemical etching of a 25 μm Pt/Ir wire immersed in a CaCl_2_ electrolytic solution (CaCl_2_ 5 g, H_2_O 30 mL, acetone 2 mL). An alternating current (AC) voltage (50 Hz, 1–10 V) is applied between the tip and a gold ring electrode with the etching solution in suspension resulting in a sharp tip, which serves as the probe. In a second step, the etched Pt/Ir tip is washed with isopropanol, then by Milli-Q water and dried with pure nitrogen gas. In the third step, the etched tip is cut to length and glued on by hand with silver epoxy to the free end of the tuning fork prong. Tip lengths are cut to approximately 200 μm to prevent unwanted potential lateral tip displacement and vibration modes that might occur for tip heights longer than 400 μm [22]. A Pt/Ir tip is chosen such that it does not oxidize in reaction conditions.

### Electronics and data acquisition

The tunneling current is collected using a preamplifier (DLPCA-200 preamp, Femto Messtechnik GmbH, Germany) with the bias applied to the sample. The tuning fork is driven by an AC voltage, and its deflection is measured by the resultant current. The qPlus signal preamplification is provided by a Femto HQA-15M-10T high-frequency charge amplifier with a high gain of 10 V/pC, before it is fed to the signal analyzer. The tip motion and the feedback loop are controlled by electronics from RHK technology. A phase-locked loop is employed for locking the phase between the AC drive signal to the QTF and the signal input. When the phase is locked, the resonance frequency of the tuning fork will shift as the tip interacts with the surface. Amplitude, frequency, and phase are measured. These signals are then fed into the electronics for monitoring and feedback options. The RHK software (R9 plus) allows for various user controls, that is, setpoint (frequency shift for AFM feedback or current for STM feedback), amplitude, and phase as well as the current images can be recorded separately or in several combined modes and compared in real time.

## Results and Discussion

To demonstrate the capabilities of the combined ReactorAFM/STM, we show results from two different types of experiments. In the first, we show operando combined AFM/STM images of a clean Pd(100) single crystal that undergoes oxidation of the surface. The oxidation happens at 450 K under 0.5 bar of oxygen atmosphere. The second experiment is a FTS experiment, where we show AFM images of catalytic cobalt nanoparticles. The nanoparticles are deposited on an oxide layer representing realistic and relevant industrial catalysts. Here, the reaction conditions exceed the temperature limit of the qPlus sensor used in that measurement. Therefore, we show AFM images taken below the reaction temperature, before and after the reaction takes place, with QMS data of the product gases during reaction. In this experiment, the substrate is an aluminum oxide layer through which electrons cannot tunnel and, thus, cannot be studied by STM methods. Before presenting these high-temperature, high-pressure experiments, we show the temperature dependence of the qPlus sensor used in these experiments.

**Figure 4:**
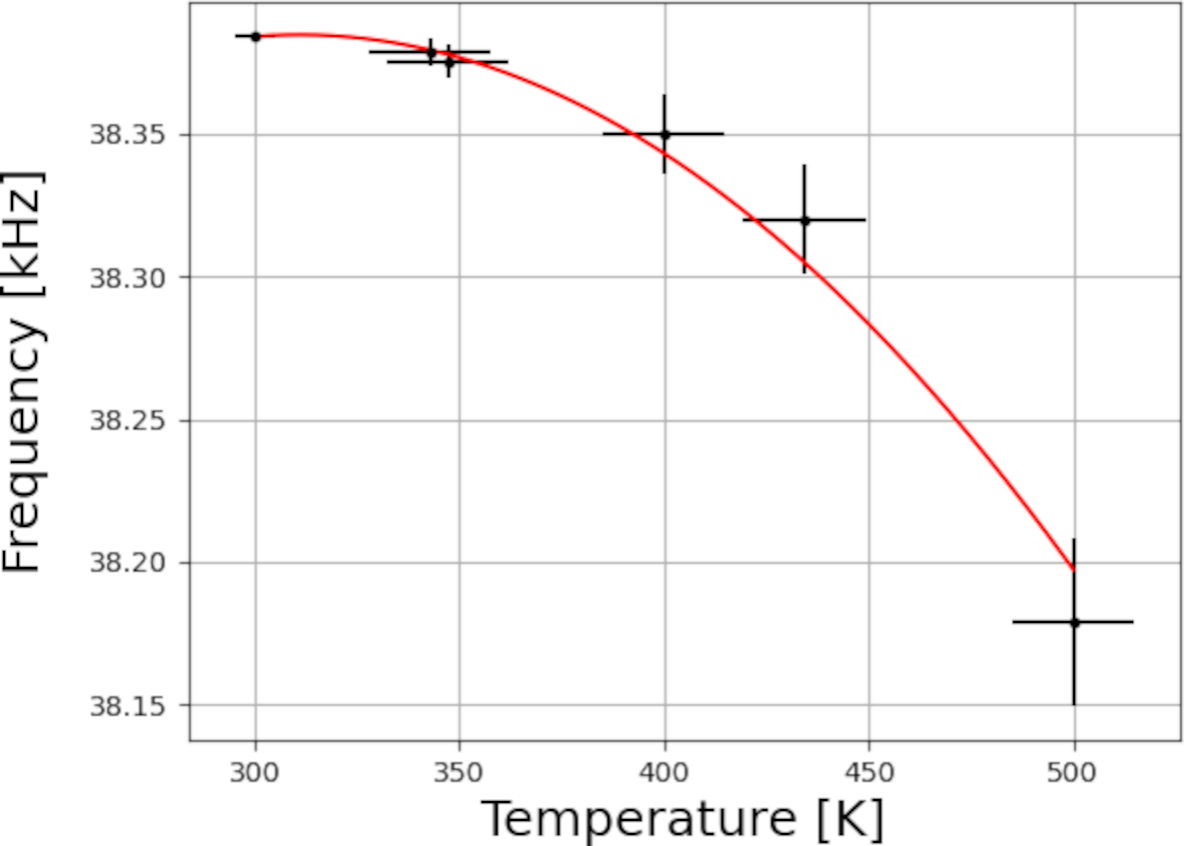
qPlus resonance frequency (black dots) as a function of the temperature ranging from room temperature to 500 K. The red curve is a fit corresponding to Equation 1 with fit parameters *f*_0_ = 38.38 ± 0.02 kHz, *c* = 0.018 ± 0.002 K^−2^, and *T*_0_ = 330 ± 10 K. Error bars in the *x* direction are based on an estimated temperature uncertainty of 15 K (except at room temperature). Error bars in the *y* direction are calculated by error propagation from the temperature uncertainty. The slope of the function at a given temperature indicates the feedback’s sensitivity to temperature fluctuations.

Figure 4 shows the resonance frequency of the qPlus sensor as a function of the temperature. The thermocouple used to measure this temperature is intended to indicate the substrate temperature on the sample holder. This means that it is situated at a certain distance from the qPlus sensor and that we cannot be certain of the sensor’s exact temperature. For this reason, we attribute a rough 15 K temperature uncertainty to these measurements, which is represented in the *x* error bars. Typically, after increasing the temperature, a minimum wait of 4 h is required for the resonance frequency to stabilize because of temperature fluctuations. The error bars in the *y* direction are calculated by error propagation on the temperature uncertainty to demonstrate the spread in resonance frequency shifts while the temperature stabilizes. The purpose of this graph is to determine the temperature range at which we can operate the tuning fork and to illustrate the degree of difficulty while scanning at high temperature. Beyond 500 K, no resonance was measured for this specific sensor as the signal became too weak. The fitted function follows Equation 1 with fitting parameters *f*_0_ = 38.38 ± 0.02 kHz, *c* = 0.018 ± 0.002 K^−2^, and *T*_0_ = 330 ± 10 K. The slope of the curve at a given temperature indicates the sensor’s sensitivity to temperature fluctuations. Near room temperature, where the slope is 0, it is relatively insensitive to temperature fluctuations, while at increasing (and decreasing [23]) temperatures, the slope is steeper; hence, it becomes more important to have a stable temperature while scanning. The data in this graph were collected under UHV conditions; increasing the pressure will affect the Q-factor [19] and, in turn, the signal-to-noise ratio.

**Figure 5:**
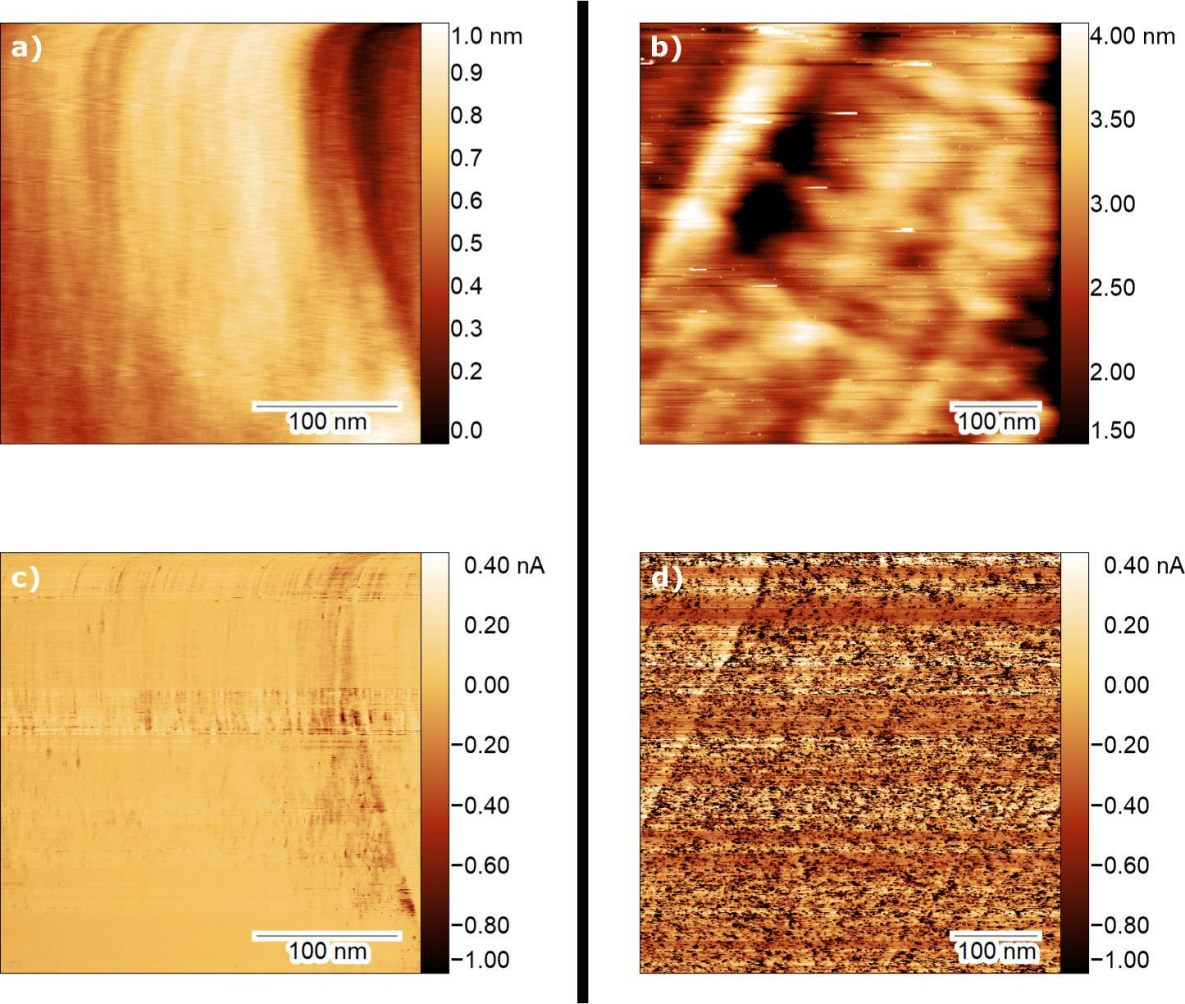
Combined AFM/STM images taken with the ReactorAFM/STM of a Pd(100) single crystal in UHV and at 450 K before reaction (a, c) and during oxidation (b, d). The top two images are topography images, and the bottom two images show the simultaneous current signal. (a) and (c) were taken under UHV conditions with d*F* = 7 Hz and bias voltage of −500 mV applied to the sample. (b) and (d) show the surface under 0.5 bar of O_2_ pressure and were taken with d*F* = 5 Hz and bias voltage of −1 V applied to the sample. RMS surface roughness and RMS current for each image are, correspondingly, (a) 0.17 nm, (b) 50 pA, (c) 0.63 nm, and (d) 760 pA.

To demonstrate the performance of the AFM/STM reactor, we show in Figure 5 images of an as-prepared Pd(100) single crystal, taken at 450 K under UHV conditions (Figure 5a) and under oxidation reaction conditions (Figure 5b). Scanning at high temperature and pressure is performed with the same feedback settings as in UHV and at RT; however, long waiting times are required and feedback might be lost because of the higher sensitivity to fluctuations in pressure and temperature while scanning. The images were taken in combined nc-AFM/STM mode using the frequency shift d*F* as feedback, while recording the tunneling current simultaneously (Figure 5c,d). Consequently, the tip–sample distance will be maintained throughout the image while the current signal will be a direct indication of the conductivity of the surface. The Pd(100) surface has been prepared using the standard recipe of repeated cycles of Ar-ion sputtering (3 μA, 1 kV, 30 min) at room temperature followed by annealing at 1000 K for 5 min.

In Figure 5a,c, taken at 450 K under UHV conditions, we recognize steps in the vertical direction, which correspond to the Pd(100) steps. In the current signal image, the same steps are visible and defined more distinctly than in the topographic image. After the introduction of 0.5 bar oxygen in the reactor, and once the qPlus sensor had stabilized (with a 30 Hz drop in resonance frequency), the tip was re-approached to the surface and scanning was started, as shown in Figure 5b with the corresponding current signal in Figure 5d. At a different field of view, we observe Pd(100) steps angled slightly off the vertical direction and less well-defined than under UHV conditions. Most notably is the appearance of high-density, insulating islands illustrated in the current signal image (appearing as dark spots with the same color contrast as in Figure 5c). By observing the root-mean-squared (RMS) surface roughness (*R*_q_) under UHV conditions of 0.17 nm in topography and 50 pA in the current signal, we determine that the surface is rather smooth and flat. In contrast, under oxidation conditions, the surface roughness in topography is increased to 0.63 nm because of the more challenging scanning conditions. However, the current signal surface roughness increases by an order of magnitude to 760 pA with respect to Figure 5c. This significant increase in surface roughness, which can be observed as the appearance of dark spots, is due to the formation of islands with reduced conductivity, which we attribute to oxidation of the surface.

**Figure 6:**
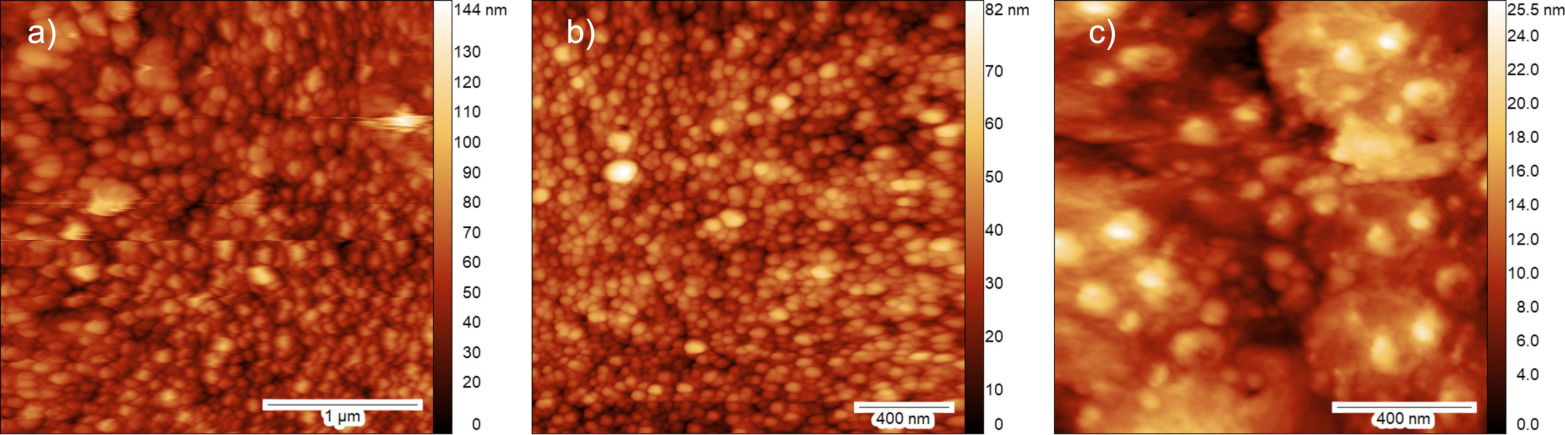
AFM images of cobalt nanoparticles on a thick (50 nm) Al_2_O_3_ film taken (a) before reaction, at RT and high vacuum, (b) at 430 K and 2 bar CO and H_2_ pressure before reaction, and (c) after reaction has occurred, at 430 K and 2 bar pressure of CO and H_2_. (a) and (c) were taken in constant signal mode of the RHK controller with setpoint 40 and 58 pA respectively, (b) is taken in constant drive mode with setpoint d*F* = 3 Hz.

To further demonstrate the ReactorAFM/STM capabilities, we show results from a FTS investigation. FTS is a series of reactions where CO and H_2_ gas react to form various hydrocarbons C*_n_*H*_2n+2_*, with water as byproduct [24]. We have investigated the reaction on Co nanoparticles deposited on an Al_2_O_3_ support, grown on a NiAl(110) single crystal. The NiAl(110) surface has been prepared by repeated cycles of Ar-ion sputtering (3 μA, 1 kV, 30 min) at room temperature followed by annealing at 1000 K for 5 min. The oxide is deposited ex situ (in a nearby setup, transfer is done in air) by physical vapor deposition using an aluminum oxide sputter target and NiAl(110) as substrate. The deposition was performed at a 10^−3^ mbar argon pressure for a duration of 40 min. Once placed back in the main setup, the sample is annealed at 800 K in 10^−6^ mbar of O_2_ to remove carbon impurities and replenish the oxygen in the oxide layer. The composition of the surface was verified by AES (not shown here). The cobalt nanoparticles were deposited by e-beam evaporation with a Co rod, an emission current of 6 mA, and beam energy of 2 kV for 7 min. Figure 6a shows an AFM image at room temperature and high vacuum (≈10^−7^ mbar) of the prepared surface in the closed reactor. The Co nanoparticles appear as bright dots on the surface with an average diameter of ≈20 nm.

Figure 6b shows the surface at 430 K under 2 bar of CO and H_2_. Due to significant changes in temperature, the tip had to be retracted and re-approached after recording the image in Figure 6a, which means that we have a new field of view. We observe that the surface otherwise looks similar to Figure 6a with respect to the density and size distribution of the particles. While maintaining the gas flow, the sample’s temperature was increased with a rate of 1 K per 10 s up to 550 K; at this temperature FTS takes place. This is outside the possible temperature window of scanning with our tuning fork, as explained above. Therefore, we were only able to scan at 430 K before (Figure 6b) and after the reaction occurred (Figure 6c). As can be seen, the surface has undergone a change due to the reaction. The particle size distribution has changed; it appears that smaller particles are no longer visible and that there is an increase in larger particle sizes.

**Figure 7:**
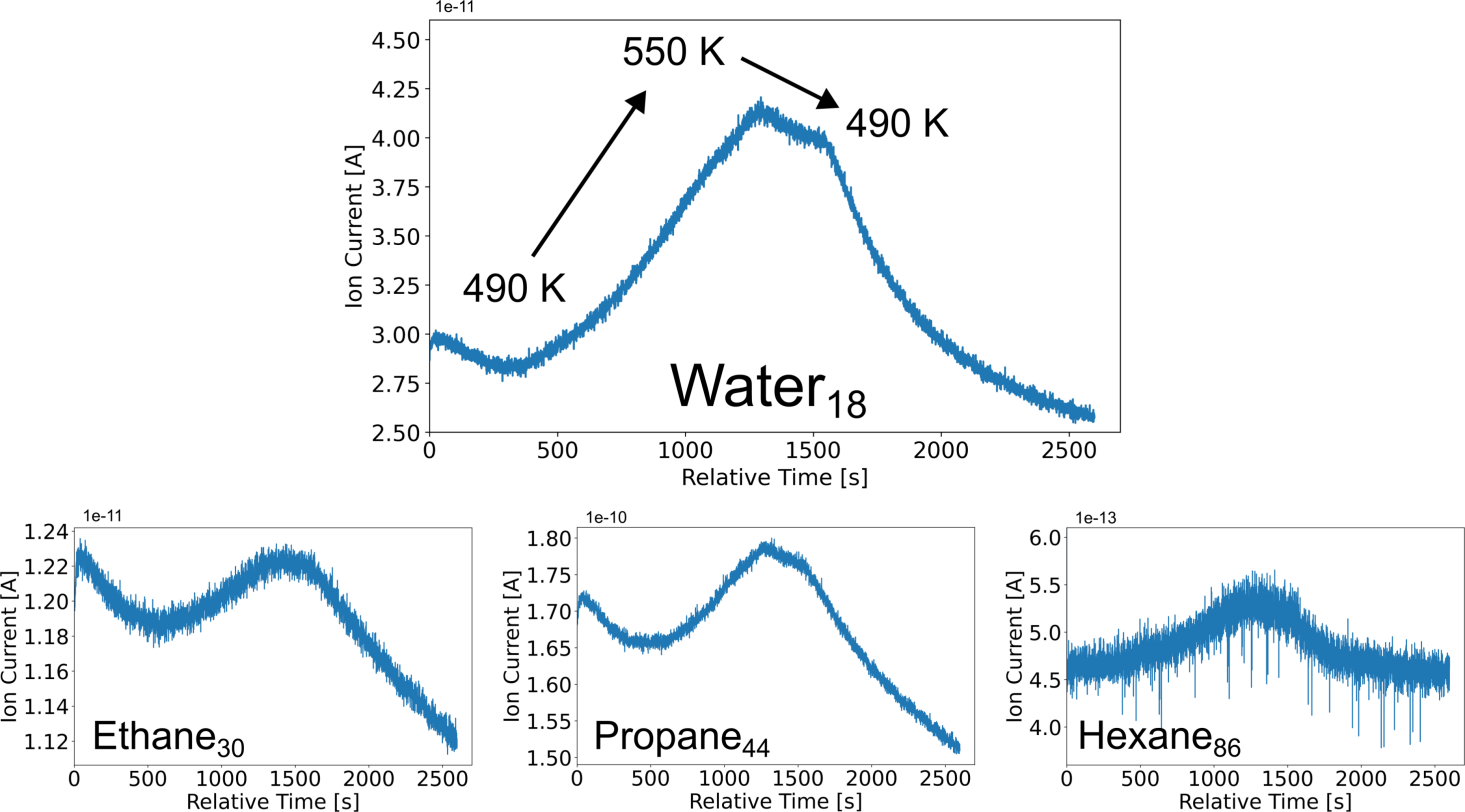
Operando QMS data of various product gases as a function of time during heating and cooling cobalt nanoparticles on a 50 nm thick Al_2_O_3_ film. Data shown for masses of *m*/*z* 18, 30, 44, and 86, which correspond to water (as byproduct), ethane, propane, and hexane, respectively.

Despite not being able to image the surface during the FTS reaction at 550 K, it was possible to measure possible reaction products using QMS at higher temperatures, as shown in Figure 7. During the heating process, at around 490 K, we observe a strong increase in the counts of water (*m*/*z* 18) indicating that the FTS reaction has started. After ≈1200 seconds, the water signal reaches a maximum. Hereafter, the temperature decreases, until reaching 490 K, where the reaction completely stops and the number of counts starts to significantly decrease. At 490 K, at which point the Co catalyst ceases its reactivity, the reaction stops. Furthermore, we observe maxima in masses of *m*/*z* 30, 44, and 86, representing ethane, propane, and hexane, respectively, in the same time frame as that for the observation of water formation.

## Conclusion

The developments in the ReactorAFM/STM design prove it to be an instrumental tool to study (catalyst) materials under industrially relevant conditions. We show that at present, we can operate the qPlus sensor at temperatures of up to 500 K while exposing the surface to reactive gases of a few bars. With combined AFM/STM, we were able to visualize the electronic structure changes of the surface under reaction conditions. Variations in the root-mean-squared current signal verifies that the surface is undergoing oxidation. Furthermore, with an industrially relevant example of cobalt nanoparticles on an oxide support, we were able to image the catalyst before and after reaction at high temperature and pressure. Quadrupole mass spectrometry data of H_2_O as byproduct and the hydrocarbons ethane (*m*/*z* 30), propane (*m*/*z* 44), and hexane (*m*/*z* 86), confirmed that Fischer–Tropsch synthesis has occurred and demonstrated the abilities of the setup.

Further developments of the ReactorAFM/STM could benefit from increasing the temperature range of the quartz tuning forks. This could be achieved by further investigating the temperature limitation and considering one designed with a higher *T*_0_, offering greater stability at elevated temperatures. Additionally, exploring the effects of varying support thicknesses and materials, as well as size distributions of metallic nanoparticles, and identifying which product gases are favored under specific reaction conditions, could extend our understanding of FTS.

## Data Availability

Data generated and analyzed during this study is available from the corresponding author upon reasonable request.
